# MRI-based radiomic and machine learning for prediction of lymphovascular invasion status in breast cancer

**DOI:** 10.1186/s12880-024-01501-3

**Published:** 2024-11-27

**Authors:** Cici Zhang, Minzhi Zhong, Zhiping Liang, Jing Zhou, Kejian Wang, Jun Bu

**Affiliations:** 1 Department of Radiology, Guangzhou Red Cross Hospital, Guangzhou, GuangDong 510220 China; 2 Innovative Institute of Chinese Medicine and Pharmacy, Shandong University of Traditional Chinese Medicine, Jinan, Shandong 250300 China

**Keywords:** Breast cancer, Lymphovascular invasion, Machine learning, Radiomics

## Abstract

**Objective:**

Lymphovascular invasion (LVI) is critical for the effective treatment and prognosis of breast cancer (BC). This study aimed to investigate the value of eight machine learning models based on MRI radiomic features for the preoperative prediction of LVI status in BC.

**Methods:**

A total of 454 patients with BC with known LVI status who underwent breast MRI were enrolled and randomly assigned to the training and validation sets at a ratio of 7:3. Radiomic features were extracted from T2WI and dynamic contrast-enhanced (DCE) of MRI sequences, the optimal feature filter and LASSO algorithm were used to obtain the optimal features, and eight machine learning algorithms, including LASSO, logistic regression, random forest, k-nearest neighbor (KNN), support vector machine, gradient boosting decision tree, extreme gradient boosting, and light gradient boosting machine, were used to construct models for predicating LVI status in BC. The area under the receiver operating characteristic curve (AUC), accuracy, sensitivity, and specificity were used to evaluate the performance of the models.

**Results:**

Eighteen radiomic features were retained to construct the radiomic signature. Among the eight machine learning algorithms, the KNN model demonstrated superior performance to the other models in assessing the LVI status of patients with BC, with an accuracy of 0.696 and 0.642 in training and validation sets, respectively.

**Conclusion:**

The eight machine learning models based on MRI radiomics serve as reliable indicators for identifying LVI status, and the KNN model demonstrated superior performance.This model offers substantial clinical utility, facilitating timely intervention in invasive BC and ultimately aiming to enhance patient survival rates.

## Introduction

Breast cancer (BC) is the most common cancer in women, with increasing morbidity and mortality, making it a major public health concern worldwide [1]. Previous studies have indicated that lymphovascular invasion (LVI) is an important step in the invasion-metastasis cascade of BC; in this process, invasive malignant cells invade the surrounding stroma, penetrate the vessel wall, and complete metastatic dissemination [2, 3]. Therefore, the presence of LVI is intimately associated with recurrence and or metastasis, poor survival and prognosis [4].

Currently, invasive pathological examination is the gold standard for diagnosing LVI in patients with BC. Partial sampling from the tumor with preoperative true-cut biopsy makes it difficult to evaluate the presence of LVI; postoperative specimens are too late for treatment [5]. There is a need to explore noninvasive and quantitative alternatives for the early diagnosis of LVI. MRI is widely used for BC, and provides morphological characteristics and functional information about lesions. Our previous studies suggested that some MRI features, such as heterogeneous fibroglandular tissue, shape, and axillary lymph node size, were significantly associated with LVI [6]; these features are somewhat subjective, and more objective and reliable markers are desirable to identify LVI status in patients with BC.

Radiomics uses high-throughput extraction of high-dimensional image features from tumor lesions, objectively and quantitatively measures the pixels and their arrangement patterns in tumor lesions, and quantifies the internal lesion information of the tumor, revealing tumor features that may not be discernible with the naked eye [7]. Through image feature extraction and machine learning technology realize real-time, comprehensive, and dynamic capture of tumor heterogeneity. Furthermore, recent advancements in AI and deep learning have revolutionized medical imaging analysis. For instance, the 2MGAS-net, a novel multi-level multi-scale gated attentional squeezed network, has demonstrated remarkable accuracy in polyp segmentation [8]. Advanced machine learning and artificial intelligence techniques have been widely studied for the early detection of LVI in BC [7, 9]. Digital mammography is reported to be insufficient for predicting LVI [10]. Ultrasound, especially the elastic heterogeneity value, achieves high sensitivity but mediocre specificity [9]. MRI is the most common modality used for LVI assessment in BC; multiparametric MRI based radiomics has yielded satisfied diagnostic performance for preoperative prediction of LVI and clinical outcomes in patients with breast invasive ductal carcinoma [11].

Machine learning is subdivided into three key categories: supervised, unsupervised, and reinforcement learning [12]. Machine learning algorithms can clearly distinguish between benign and malignant tumors, which can be very helpful in diagnosing cancer in physicians [13]. Deep learning models provide real-time diagnostic support for thyroid cancer, demonstrating their potential in clinical decision making [14]. Machine learning algorithms demonstrate capability in the classification of cancer types and the assessment of cancer risk, providing decision support to oncologists. Common machine learning algorithms include support vector machines (SNM), decision trees (DT), random forest (RF), and K nearest neighbor (KNN) used for the classification of breast cancer, monitoring progression, treatment, and prediction. Using different machine learning algorithms varying performances [15], in order to achieve better predictive LVI model applications, this will require considerable refinement and testing to realize their potential in reducing the health and economic burden of breast cancer. The novelty of our work lies in the development of eight machine learning models that integrate features based MRI to achieve better predictive LVI of BC. This approach is particularly relevant in the current stage of AI and deep learning-based classification algorithms, which are increasingly being used for early disease detection, and reduce the need for invasive surgery and related risks. Early detection can lead to timely and personalized treatment plans and improve patient outcomes.

## Materials and methods

### Study design

This study was approved by the Ethics Committee of Guangzhou Red Cross Hospital (approval no. 2021-134-01). Individual informed consent was waived because this was a retrospective study. The study was performed in accordance with the 1964 declaration of HELSINKI and the later amendments.

### Patient selection

**Fig. 1:**
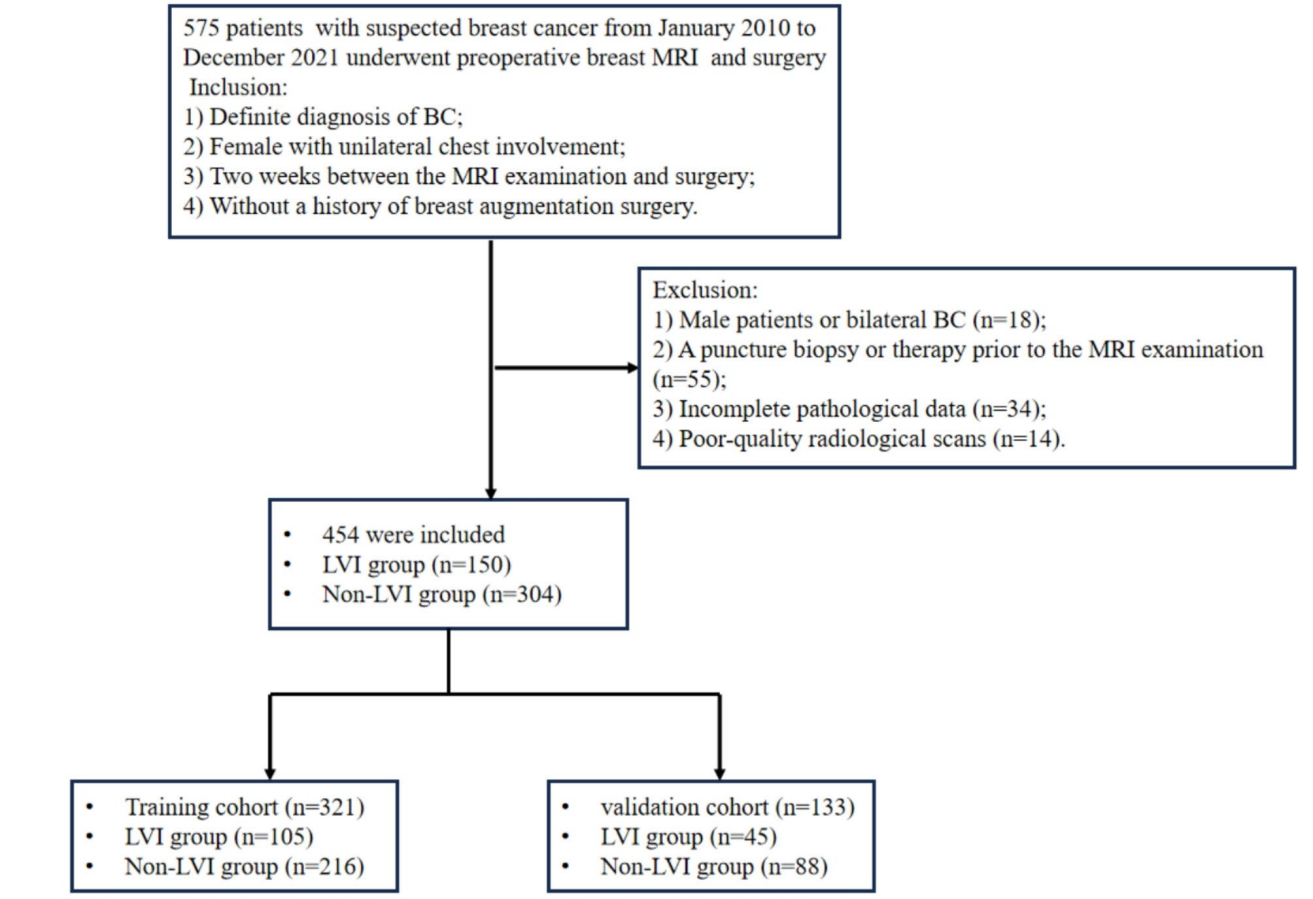
Patient selection flowchart

Patients who underwent preoperative breast MRI between January 2010 and December 2021 for BC were evaluated. Patients meeting the following criteria were included in our study: (1) histopathologically confirmed BC; (2) female with unilateral BC; (3) an interval of less than two weeks between the MRI examination and surgery; and (4) no history of breast augmentation surgery. Patients meeting the following criteria were excluded: (1) male patients or bilateral BC; (2) with a puncture biopsy, adjuvant chemotherapy, or targeted therapy prior to the MRI examination; and (3) incomplete data or poor quality of MR images. The patient selection process is shown in Fig. 1.

Histopathological examination based on postoperative specimens is the gold standard for confirming LVI in patients with BC. The presence of tumor cells in the lymphatic interstitial or vascular spaces of the peritumoral region was defined as LVI. In addition, the clinical characteristics of the patients, including age, family history of cancer, menstruation status, preoperative carbohydrate antigen 153 (CA153), and carcinoembryonic antigen (CEA), were derived from electronic medical records.

### MRI acquisition

MRI was performed using a 1.5T MRI scanner (Siemens Healthcare, Germany). T2WI and DCE (Phase II) were acquired. The contrast between the tumor and background was the largest in phase II (60–119 s) images; thus, phase II of DCE images were used as the experimental data. For the detailed specifications of the MRI parameters, please refer to Supplementary Table 1.

Radiomics Workflow.

**Fig. 2:**
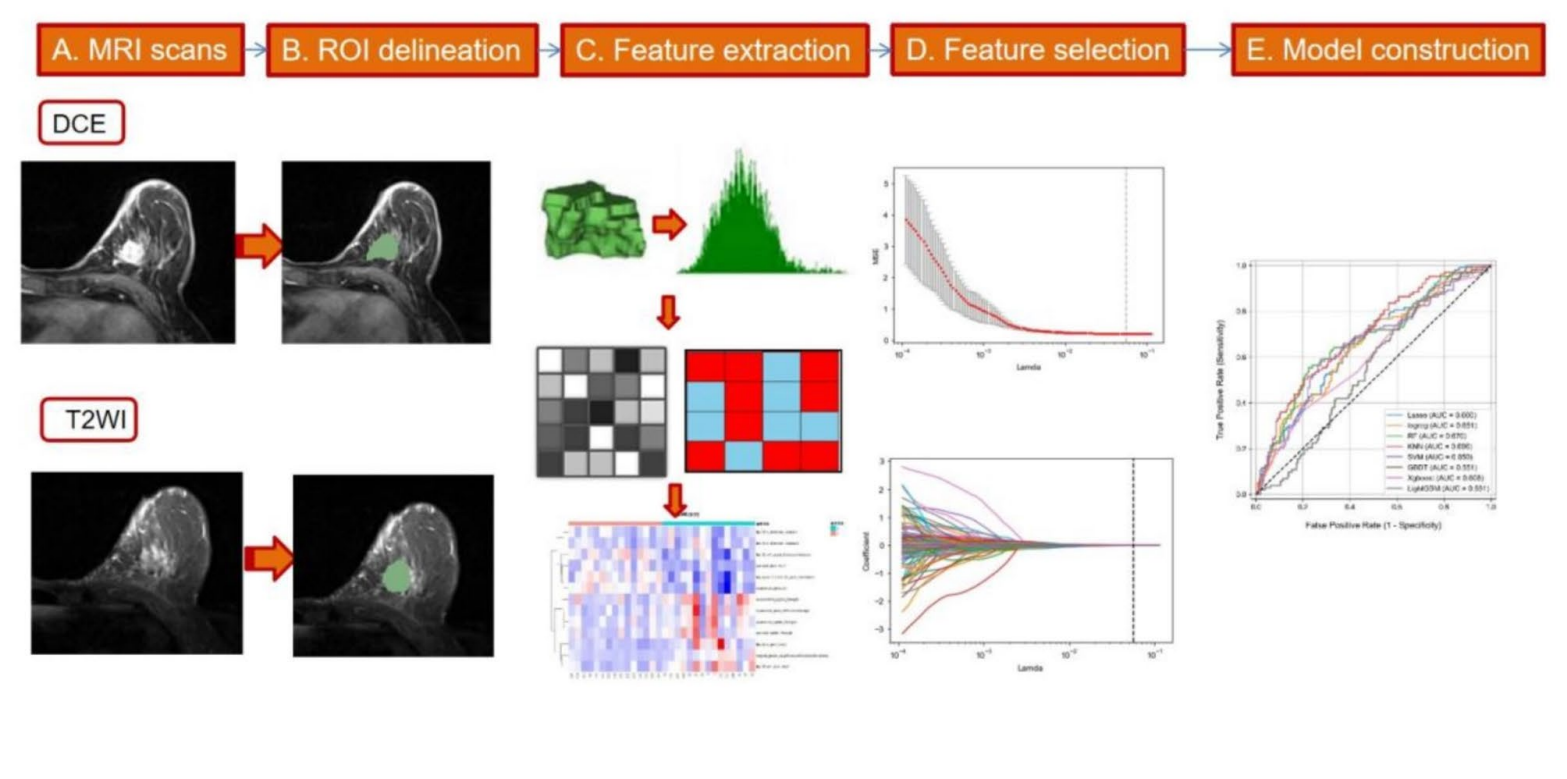
Workflow of the radiomics analysis

As shown in Fig. 2, the workflow includes (A) MRI scans, (B) ROI delineation, (C) radiomic feature extraction, (D) feature selection, and (E) model construction.

### Volume of interest (VOI) delineation

Using ITK-SANP software (http://www.itksnap.org), two experienced radiologists blinded to the pathological information of BC patients worked independently to manually delineate the VOI of the tumor layer by layer on the T2WI and DCE (phase II) sequence. The radiologists followed standardized guidelines for delineate the VOI based on the consensus protocol for breast MRI assessment. All radiologists were provided with the same standardized window and zoom settings to ensure uniformity in VOI delineation. The display parameters were pre-set and fixed for each scan, minimizing variability in how the images were viewed. The radiologists had access to the same imaging sequences, including T2WI, and DCE (phase II) sequences. The radiologists were instructed to include the entire visible tumor mass in the VOI, avoiding surrounding tissues. Disagreements regarding VOI were resolved by consensus discussions. Fifty patients were randomly selected for the second segmentation to verify the reproducibility of the feature extraction and to remove features with an intraclass correlation coefficient (ICC) below 0.8.

### Radiomics feature extraction and selection

Radiomics features were extracted from the VOIs of the MRI images using the Pyradiomics package (http://www.radiomics.io/pyradiomics.html) in the training set. A total of 3672 radiomic features were extracted from the MRI images (1836 from the T2WI sequence and 1836 from the DCE). The extracted radiomics features included first-order shape, texture, wavelet, exponential, and square transform features. Initially, the intraclass correlation coefficient (ICC1) and interclass correlation coefficient (ICC2) were calculated to assess the consistency of the lesion delineation between the two radiologists. The features with ICC1 and ICC2 < 0.8 were removed from the final dataset. Second, the z-score was deployed to standardize the radiomics features of the training set to within an identical magnitude to eliminate dimensional differences in the dataset. Subsequently, the least absolute shrinkage and selection operator (LASSO) with 10-fold cross-validation was applied for further feature selection. To ensure the reproducibility of our random assignment between training and testing cohorts, we employed a stratified k-fold cross-validation approach with a seed value to maintain consistency in our model training and evaluation process.

### Construction, validation, and performance of machine learning models

Eight common machine learning algorithms, LASSO, logistic regression (LR), RF, KNN, SVM, gradient boosting decision tree (GBDT), extreme gradient boosting (XGBoost), and Light Gradient Boosting Machine (Light GBM), were used to develop a model based on filtered radiomics features.

In the validation set, receiver operating characteristic curves (ROCs) were created to assess the diagnostic performance of radiomic signatures in distinguishing between LVI-positive BC patients and those without LVI. The area under the curve (AUC) values, accuracy, sensitivity, specificity, positive predictive value (PPV), and negative predictive value (NPV) were determined.

### Statistical analysis

The statistical analyses were conducted using R software (v4.0.5) and Python (v3.7.6). For continuous variables, significant differences were determined using the Wilcoxon rank-sum test or the t-test. The significance of differences in categorical variables was assessed using the Chi-square test. Statistical significance was set at *P* < 0.05. The interobserver reproducibility of feature extraction was evaluated using the ICC.

## Results

### Patient characteristics

A total of 454 patients, aged 26–91 years, were enrolled in our study. Patients were randomly assigned to either the training set (*n* = 321) or the validation set (*n* = 133). No statistically significant differences were observed between these groups in terms of age, menopausal status, family history of tumors, and serum levels of CEA and CA153 (Table 1).

**Table 1:** Characteristics of LVI and Non-LVI BC patients

	Total (*N* = 454)	LVI (*N* = 150)	Non-LVI (*N* = 304)	*P*-value
Age, yr, Mean (SD)	57.8 (12.6)	59.2 (12.6)	57.1 (12.6)	0.0865
Menstruation status				0.929
Premenopausal	312 (68.7%)	104 (69.3%)	208 (68.4%)	
Postmenopausal	142 (31.3%)	46 (30.7%)	96 (31.6%)	
Family history of BC, (%)	122 (26.9%)	47 (31.3%)	75 (24.7%)	0.163
CEA, µg/L, Mean (SD)	6.65 (58.0)	5.39 (13.6)	7.28 (70.2)	0.651
CA153, µg/L, Mean (SD)	20.3 (42.9)	23.3 (38.9)	18.8 (44.8)	0.266

### Radiomics features selection

**Fig. 3:**
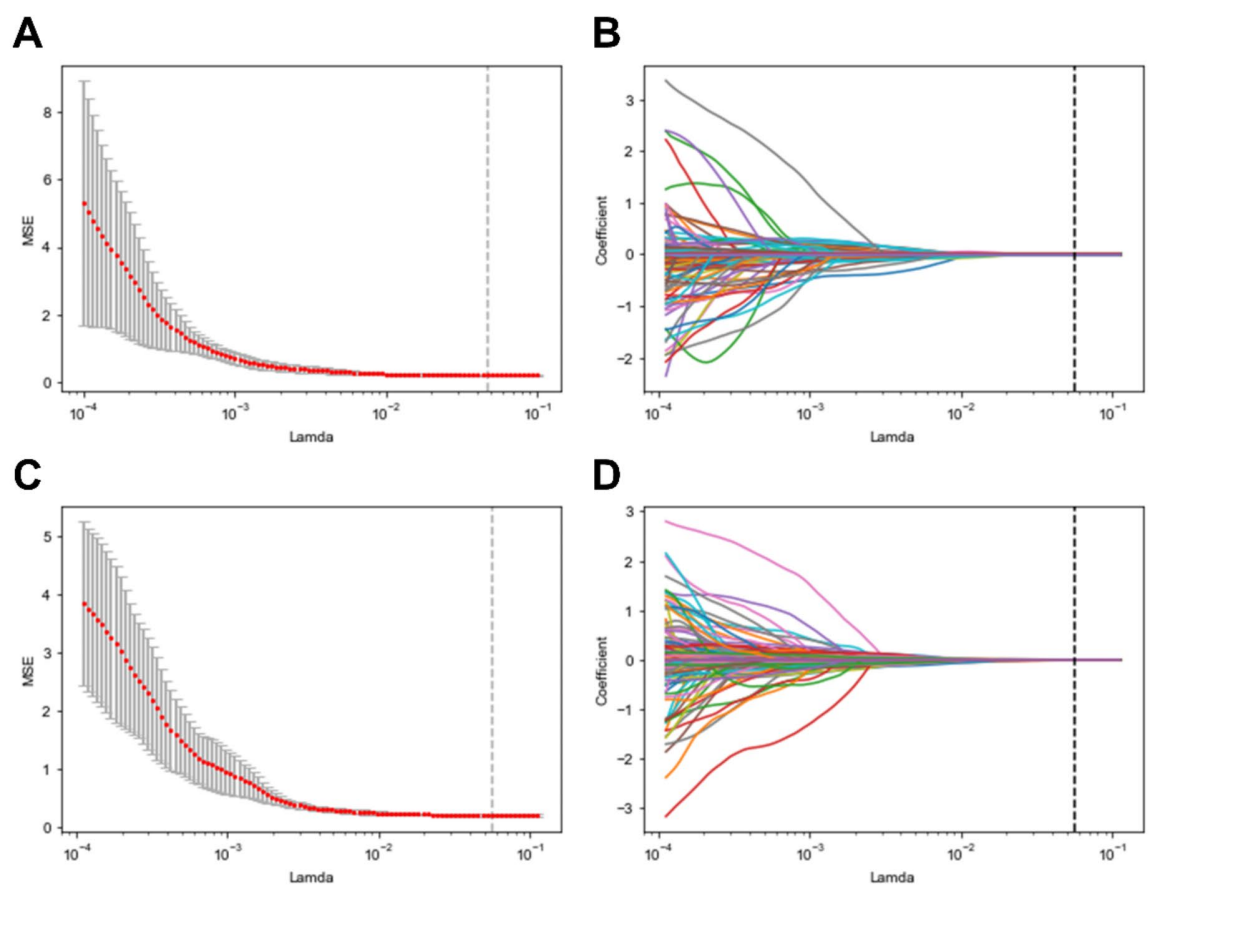
Radiomics feature selection using the LASSO algorithm. The cross-validation curves of LASSO regression analysis (A) and the LASSO coefficient path plots (B) reveal the radiomics feature selection procedure in the DCE sequence; (C). and (D) represent the radiomics feature selection procedure in the T2WI sequence

After conducting a series of filtered analyses, 18 radiomics features with good reproducibility were extracted from the primary 3672 features to construct the machine learning algorithm models (see Fig. 3). Among these 18 features, 10 were from DCE and 8 were from T2WI, as detailed in Table 2. The radiomics feature types relevant to the LVI status include first-order histogram, Gray Level Co-occurrence Matrix (GLCM), Gray Level Run Length Matrix (GLRLM), Gray Level Size Zone Matrix (GLSZM), and neighboring gray tone difference matrix (NGTDM).

**Table 2:** Radiomics features selected from DCE and T2WI sequences

Order	Sequence	Filter Type	Feature	Features Name
1	DCE	Original	GLRLM	RunVariance
2		Log-sigma-1-0-mm-3D	NGTDM	Strength
3		Wavelet-HLH	First Order	InterquartileRange
4		Wavelet-HHL	GLSZM	LargeAreaHighGrayLevelEmphasis
5	DCE	Lbp-3D-m1	GLSZM	SmallAreaHighGrayLevelEmphasis
6		Lbp-3D-k	First Order	Variance
7		Lbp-3D-k	GLCM	Imc2
8		Squareroot	GLCM	Idmn
9		Gradient	GLCM	Imc1
10		Gradient	NGTDM	Strength
11	T2WI	log-sigma-1-0-mm-3D	First Order	Skewness
12	T2WI	wavelet-HLH	First Order	Mean
13	T2WI	wavelet-HHL	ngtdm	Strength lbp-3D-
14	T2WI	m1	glrlm	LongRunLowGrayLevelEmphasis
15	T2WI	lbp-3D- m1	glszm	SizeZoneNonUniformityNormalized
16	T2WI	lbp-3D-k	glrlm	RunEntropy
17	T2WI	lbp-3D-k	glrlm	ShortRunLowGrayLevelEmphasis
18	T2WI	squareroot	First Order	Minimum

### Machine learning models construction and validation

**Fig. 4:**
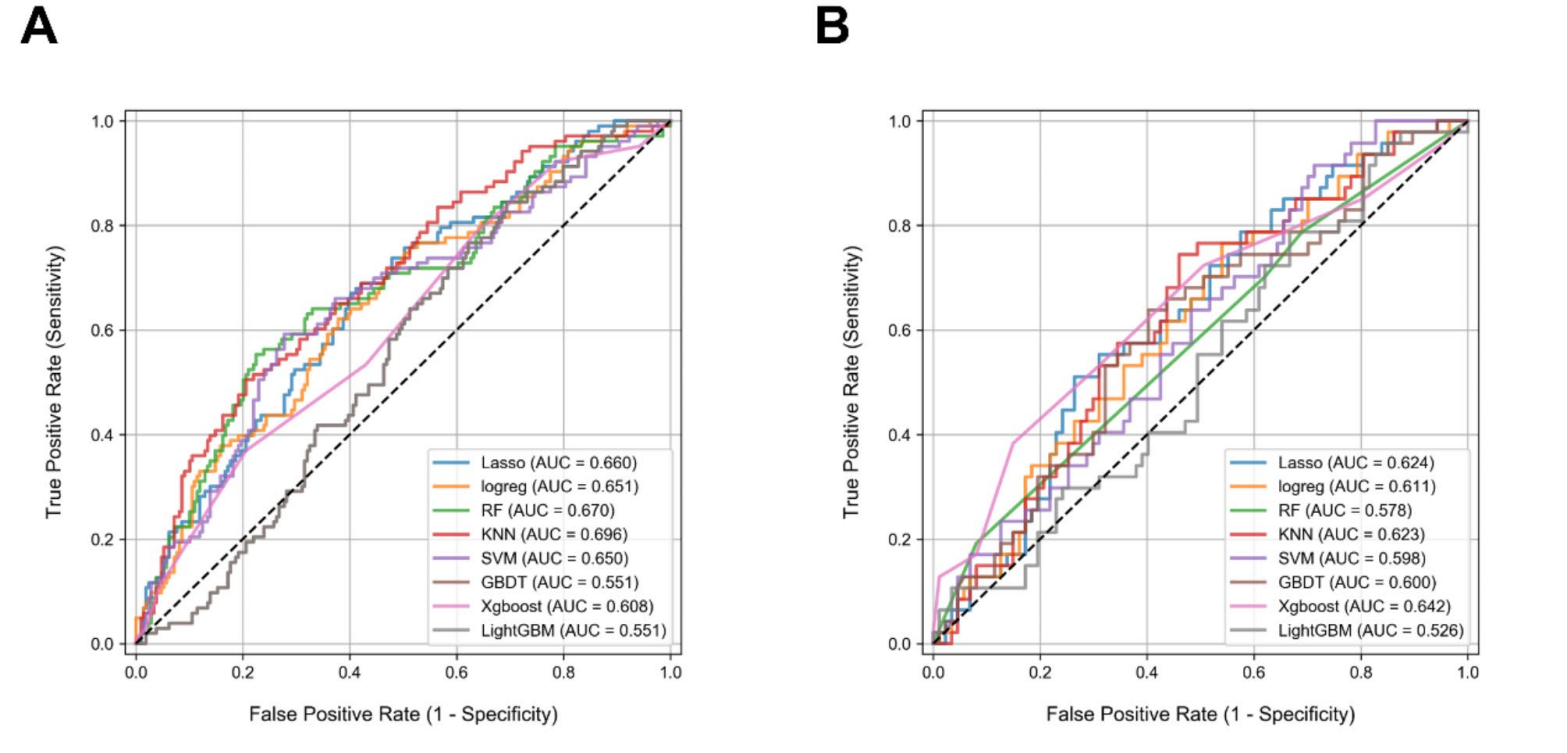
The ROC curves demonstrate the discriminatory performance of the LASSO, LR, RF, KNN, SVM, GBDT, XGBoost, and LightGBM models to predict LVI in BC patients. (A) in the training set. (B) in the validation set

Eight common machine learning algorithms to construct radiomics models, including LASSO, LR, RF, KNN, SVM, GBDT, XGBoost, and LightGBM, were used to develop a model for assessing the LVI status in BC patients. The ROC curves of these models are shown in Fig. 4. Among all the models, the KNN exhibited superior performance in predicting LVI status in BC patients with an AUC of 0.642 in predicting LVI status in BC patients. Further diagnostic performance evaluations, including sensitivity, specificity, and accuracy, are summarized in Table 3.

**Table 3:** Prediction performance of eight machine learning models in validation set

algorithm	AUC	Accuracy	NPV	PPV	Sensitivity	Specificity
Lasso	0.624	0.649	0.727	0.500	0.489	0.736
Logistic	0.611	0.560	0.769	0.427	0.745	0.460
RF	0.633	0.545	0.760	0.417	0.745	0.437
KNN	0.642	0.657	0.672	0.533	0.170	0.920
SVM	0.526	0.485	0.725	0.383	0.766	0.333
GBDT	0.628	0.619	0.790	0.472	0.723	0.563
XgBoost	0.598	0.500	0.833	0.404	0.894	0.287
LightGBM	0.600	0.604	0.743	0.453	0.617	0.598

## Discussion

In the present study, we developed and validated eight machine learning models based on MRI radiomic signatures derived from T2WI and DCE sequences to predict LVI in patients with BC. Different sequences carry distinct potential information regarding the microstructure and biological behavior of tumors, reflecting different tumor characteristics. T2WI allows clear delineation of the lesion’s size and shape, lesion intensity, and provides greater sensitivity to cystic and necrosis within the lesion. A recent study suggested an association between peritumoral edema observed on T2WI and LVI in BC [16]. A previous study showed the potential ability of DCE-MRI radiomics to non-invasively assess angiogenesis or microvessel density [17]. Moreover, DCE-MRI radiomics has been used to predict LVI in BC patients [18]. Choi et al. reported that DCE performed well in predicting LVI status in patients with node-negative invasive BC [19]. Therefore, it is reasonable to explore the combination of DCE and T2WI radiomics in the prediction of LVI. Our study found that combining T2WI and DCE sequences performed well in predicting LVI in BC, aligning with previous research. Zhang et al. [11] found that adding radiomic features extracted from T2WI images may improve the diagnostic performance of other MRI sequences, and the fusion radiomic signature of T2WI, cT1WI, and ADC maps achieved better predictive efficacy for LVI than any of these sequences alone, though the sample size was relatively small. Notably, previous clinical studies have shown inconsistent performance of clinical models based on different MRI sequences. Kayadibi et al. revealed that ADC alone performed better than cT1WI images, or cT1WI combined with ADC map modeling in predicting LVI status [20]. Nijiati M. reported that an ADC radiomic model achieved better performance, but combining radiomic features derived from T2WI, DCE, and DWI failed to provide incremental value, indicating that multiparametric MRI-derived radiomic features did not achieve a complementary effect in predicting LVI status [7]. These studies indicate that radiological features are promising indicators of LVI in patients with BC, however, further studies based on multidimensional MR imaging data are required to confirm the different sequences in LVI diagnosis in BC.

Using computer vision and radiomics, we successfully extracted various imperceptible radiological features, including histogram and texture feature parameters from T2WI and DCE sequences. Sphericity, a morphological feature, has been reported as a predictor of LVI in other solid tumors; lower sphericity values imply greater deviation from a perfect sphere, indicating irregularities [21]. Our previous studies have shown that LVI-positive tumors tend to exhibit more frequent tumor morphology irregularities due to increased aggressiveness and intercellular growth rate differences [6]. Skewness, a histogram-based first-order statistic, was the parameter that attempted to quantify neoplastic heterogeneity by taking into account the asymmetry of the average grayscale distribution. Li H et al. studies have shown that the higher asymmetry in the frequency of grayscale distribution suggested higher tumor heterogeneity between the LVI-positive and the LVI-negative tumors attributed to a discrepancy in cell proliferation time, necrosis, microcalcifications [22]. The matrix-based texture feature parameters were utilized to measure the grayscale dispersion degree and texture roughness of distinct tumor regions, with increasing values corresponding to rougher texture, which indicated stronger radiomic heterogeneity of the tumor. Furthermore, the high-order features of wavelet transform also offered the potential insight to quantify tumor biological and multidimensional perspectives heterogeneity. This extensive radiological feature collection offers an excellent opportunity to investigate the relationship between these features and tumor manifestations. However, further exploration is required to interpret the association between these complex parameters and tumor biology.

Machine learning plays a crucial role in radiomic analysis, and different algorithms vary in their predictive performance. To achieve optimal predictive performance, we evaluated eight machine learning models, and the result show that the KNN algorithm outperforming than others. KNN is a simple, intuitive machine learning method used for classification and regression. It works by identifying the “k” closest neighbors to a given data point and making predictions based on the majority class for classification or the average value for regression. Its key advantages are that it is non-parametric, meaning it makes no assumptions about data distribution, and it adapts well to complex datasets with multiple classes or irregular boundaries. KNN has been successfully applied in breast cancer diagnosis and classifies based on radiomic or histopathological features [23]. Jiang Y et al. constructed models using machine learning approaches, including logistic regression, SVM, DT, KNN, and GBM, to preoperatively differentiate LVI in clinically node-negative BC, with the GBM model showing superior predictive performance and robustness [24]. Liu et al. [25] established three machine learning models (SVM, LR, and XGBoost) based on DCE-MRI radiomic features to predict sentinel lymph node metastasis in BC patients, with SVM exhibiting the best predictive performance. Similarly, Zhu et al. [26]. developed five machine learning models based on CE-MRI radiomic features for preoperative SLN metastasis prediction, where SVM, RF, LR, and GBDT models showed high AUC values, and DT was prone to overfitting, SVM excels in balancing model complexity and learning ability to achieve optimal generalization in limited sample settings. These studies used the radiomic features extracted by DCE-MRI to build different machine learning models, and achieved high predictive performance.

Utilizing the vast array of detailed and comprehensive radiological information, radiomics-based algorithmic models hold great promise in the diagnosis and prognosis assessment of BC. The development of radiomics and the application of novel imaging tools are expected to yield intricate insights into the underlying mechanisms of tumorigenesis. By unveiling the intricacies of tumor heterogeneity and the microenvironment through advanced imaging techniques, a wealth of detailed and diverse information can be obtained. This, in turn, facilitates the exploration of tumorigenesis at a deeper level [27, 28]. Moreover, the future incorporation of radiomics into a multi-omics framework holds great promise in providing profound and comprehensive insights into the intricate biological features of tumors. This integration is anticipated to be foundational for precise diagnosis and personalized treatment strategies in tumor patients [29, 30].

Our study is subject to certain limitations that must be acknowledged. Firstly, as a retrospective study, there is an inherent risk of sample selection bias. Second, the generalizability of our findings needs validation in larger sample sizes and multi-center studies. Furthermore, it is important to note that our model lacks inclusion of crucial clinical features such as pathological grading and molecular typing. Incorporating these clinical indicators into our model in future investigations is imperative in order to enhance the comprehensiveness and robustness of the model.

## Conclusions

The eight-machine learning models based on MRI radiomics serve as reliable indicators for identifying LVI status, with the RF classifier demonstrating superior performance.

## Data Availability

Data is provided within the manuscript or supplementary information files.

## Abbreviations

LVI: Lymphovascular invasion
BC: Breast cancer
MRI: Magnetic resonance imaging
DCE: Dynamic contrast-enhanced
CA153: Carbohydrate antigen 153
CEA: Carcinoembryonic antigen
LR: Logistic regression
DT: Decision trees
RF: Random forest
KNN: k-nearest neighbor
SVM: Support vector machine
GBDT: Gradient boosting decision tree
XGBoost: Extreme gradient boosting
Light GBM: Light Gradient Boosting Machine
CI: Confidence intervals
PLR: Positive likelihood ratio
NLR: Negative likelihood ratio
